# Bilateral Pretibial Varices with Intraosseous Venous Drainage Anomaly: A Case Report

**DOI:** 10.5334/jbr-btr.882

**Published:** 2015-12-30

**Authors:** F. Dermesropian, V. Scavée, J-P. Haxhe, A. Bodart, T. Puttemans

**Affiliations:** 1 Department of Radiology, Clinique Saint-Pierre, Ottignies, Belgium; 2 Department of Vascular and Thoracic Surgery, Clinique Saint-Pierre, Ottignies, Belgium

**Keywords:** Pretibial varices, intraosseous venous drainage, tibia

## Abstract

We report the case of a 35-year-old male patient who complained of right anteromedial leg pain, after an intensive sport exercise. At physical examination, internal pretibial soft tissue swelling containing prominent painful varices was found. Color Doppler ultrasound, radiographic examinations, followed by CT and MR complementary investigation, were performed.

## Clinical history

We report the case of a 35-year-old male patient who complained of right, anteromedial leg pain after an intensive sport exercise. At physical examination, internal pretibial soft tissue swelling containing prominent painful varices was found. Color-Doppler ultrasound and radiographic examinations, followed by computed tomography (CT) and MR complementary investigations, were performed.

## Image findings

**Figure 1:**
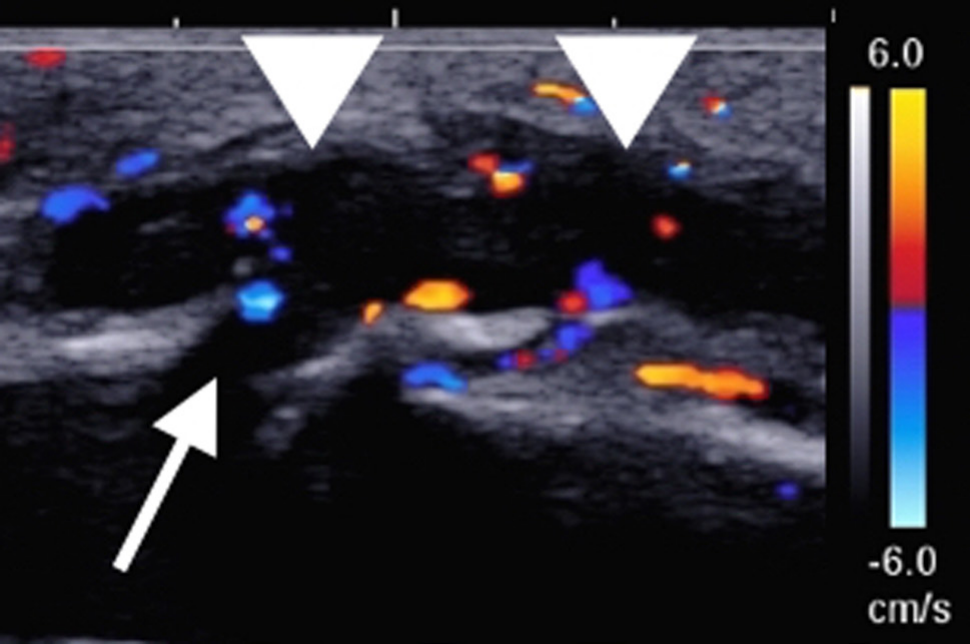
Sagittal B-mode and color-Doppler ultrasound images show thrombosed varices (*arrowheads*) located anteriorly to the tibial shaft, coursing posteriorly through a cortical defect (*arrow*).

Color-Doppler sonography revealed a plot of thrombosed pretibial varices. A venous course was found through a focal cortical defect of the anteromedial tibial cortex (Figure 1).

**Figure 2:**
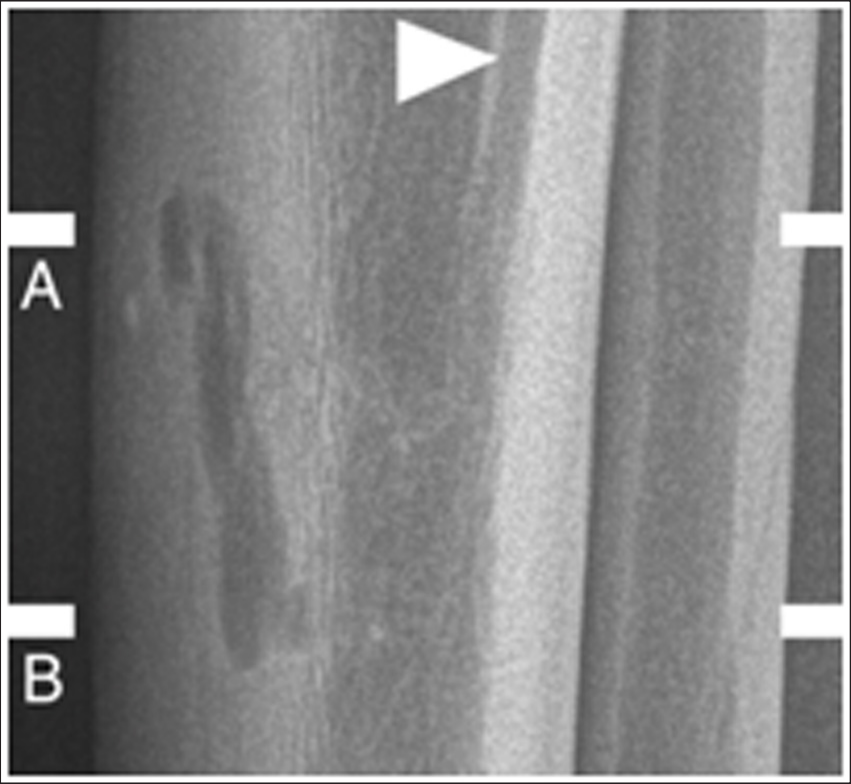
Lateral radiographs of the right lower leg show a small, ill-defined subcortical round lucency in the midshaft of the anterior tibial cortex and a prominent vascular groove (*arrowhead*).

Conventional radiography showed a small subcortical osteolytic defect immediately beneath the pretibial varices, and an enlarged nutrient canal in the posterior tibial diaphysis (Figure 2).

**Figures 3 and 4:**
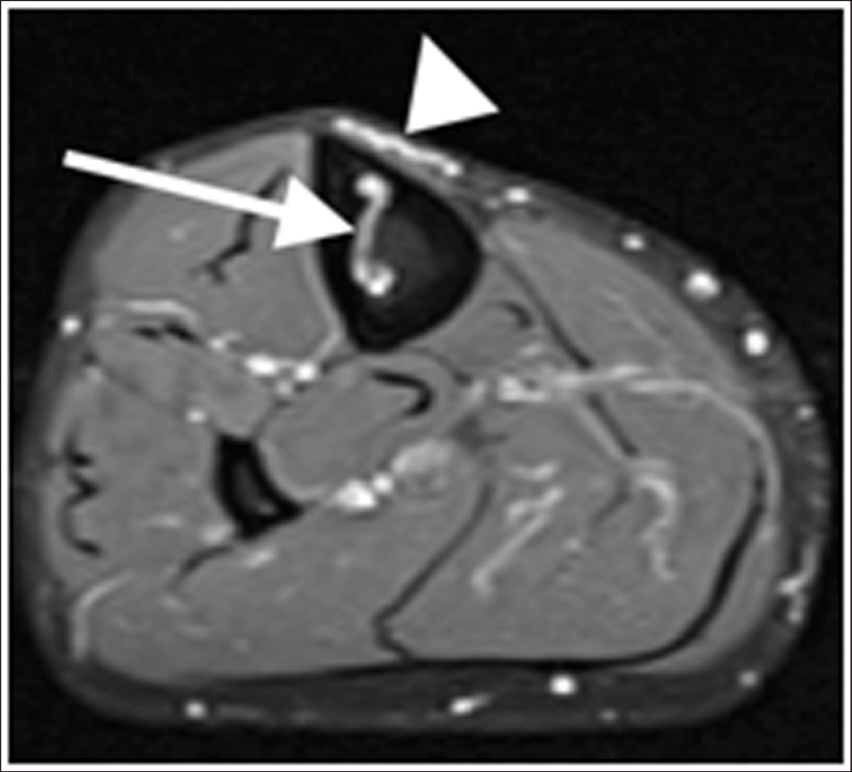
Axial CT-scan images (3 and 4, respectively, right and left leg) show a serpiginous tubular structure (*arrowhead*s) extending from the cortical defect (subjacent to the pretibial varices), coursing intramedullary as an intraosseous vein and entering a vascular groove (*arrows*) in the posterior cortex of the proximal tibial diaphysis. The diameter of the nutrient groove was enlarged bilaterally.

CT images (Figure 3 and 4) confirmed the presence of a focal cortical defect in the middle one-third of the right tibial diaphysis, subjacent to the pretibial varices. A serpiginous tubular structure extended from this defect, coursed intramedullary as an enlarged intraosseous vein and entered an enlarged vascular groove in the posterior cortex of the proximal tibial diaphysis.

**Figure 5:**
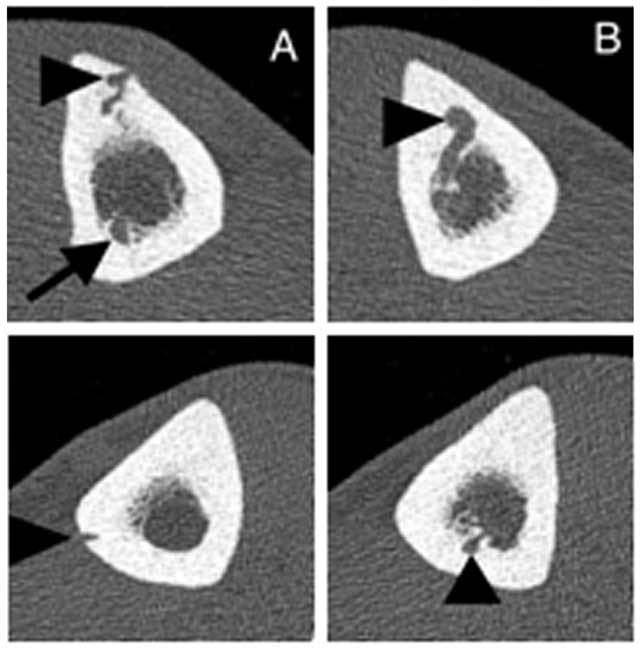
Axial T1-weighted with fat saturation after gadolinium injection MR imaging of the right midtibial diaphysis demonstrates pretibial varices (*arrowheads*), one of which travels through the anterior tibial cortex to an enlarged intraosseous vein (*arrow*) and prominent nutrient vascular canal.

These findings were also present on the asymptomatic contralateral tibia. MR examination showed no signal intensity alteration within the adjacent muscular structures (Figure 5).

## Discussion

Varicose veins in lower extremities affect about 15% of world population.

The anomalous communication between pretibial varices and an intraosseous tibial vein presented by our patient is a rare condition that has been reported a few times in the literature, and to our knowledge, the first reported case was bilateral. The physiopathology is not fully understood. Some authors hypothesize that intraosseous venous drainage anomaly may be the cause of varices or deep venous thrombosis and conversely the consequence of venous insufficiency [1].

The diagnosis can be done using imaging alone. Color-Doppler ultrasound confirms the presence of the characteristic venous flow pattern in the varices through a cortical impression defect. CT and MRI are used to confirm the diagnosis, to exclude the presence of soft tissue or osseous mass and to rule out other vascular anomalies [2]. Differential diagnoses include hemangiomas, arteriovenous malformation and venous malformations. Ambulatory phlebectomy, ligation and stripping, or percutaneous ablation, are the usual treatment options. Sclerotherapy is not considered because of the intraosseous communication of the varix.

## Conclusion

Diagnosis of pretibial varices with intraosseous venous drainage anomaly can be made by using imaging alone. Recognition of this rare anomaly can prevent misdiagnosis, and it is pivotal in the planning of appropriate treatment.

## Competing Interests

The authors declare that they have no competing interests.
